# Ecology of Powassan Virus in the United States

**DOI:** 10.3390/microorganisms9112317

**Published:** 2021-11-09

**Authors:** Erin M. Hassett, Saravanan Thangamani

**Affiliations:** SUNY Center for Vector-Borne Diseases, Department of Microbiology and Immunology, Institute for Global Health and Translational Science, Upstate Medical University, Syracuse, NY 13210, USA; HassettE@upstate.edu

**Keywords:** Powassan virus, deer tick virus, ticks, tick-borne virus

## Abstract

Zoonotic viruses threaten the lives of millions of people annually, exacerbated by climate change, human encroachment into wildlife habitats, and habitat destruction. The Powassan virus (POWV) is a rare tick-borne virus that can cause severe neurological damage and death, and the incidence of the associated disease (Powassan virus disease) is increasing in the eastern United States. The mechanisms by which POWV is maintained in nature and transmitted to humans are complex and only partly understood. This review provides an overview of what is known about the vector species, vector-host transmission dynamics, and environmental and human-driven factors that may be aiding the spread of both the vector and virus.

## 1. Introduction

Emerging and re-emerging zoonotic viruses pose an increasing threat to human and animal health, as demonstrated by the recent SARS CoV-2 pandemic which has resulted in 190 million human cases and more than 5 million deaths worldwide [1]. Climate change, increased human encroachment into wildlife habitats, and habitat fragmentation contribute to the expanding distribution of disease vectors, concentration of hosts (uninfected and infected), and, consequently, increased contact between host and disease vectors [2,3,4]. Ticks are particular vectors of concern since approximately 95% of all arthropod-borne diseases in the United States (US) are caused by tick-borne pathogens [5]. Notably, Lyme disease, caused by the bacterium *Borrelia burgdorferi*, is the most prevalent vector-borne disease in the US, resulting in an estimated 476,000 cases a year [6]. However, tick-borne pathogens rarer and deadlier than *B. burgdorferi* are rising in prevalence in the vector population, namely the Powassan virus (POWV) [7].

**Figure 1:**
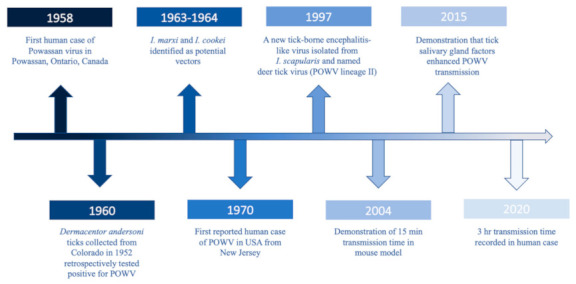
Timeline of major POWV milestones.

POWV is a neuroinvasive, single-stranded, positive sense RNA tick-borne flavivirus (*Flaviviridae*), and it is the only member of the tick-borne encephalitis serogroup in North America. POWV was first isolated in 1958 from the brain of a five-year-old boy from Powassan, Ontario who died of encephalitis (Figure 1) [8]. In 1970, the first human case of POWV in the US was reported in New Jersey (Figure 1) [9]. Approximately two decades later, a tick-borne encephalitis-like virus was detected in *Ixodes scapularis* (deer ticks) which was genetically different than POWV and thus was named deer tick virus (Figure 1) [10]. Subsequently, it was discovered that POWV had two lineages: Powassan virus lineage I (POWV-LB), isolated from the first case, and Powassan virus lineage II (DTV) isolated from *I. scapularis* ticks [10]. While the two lineages share 84% nucleotide identity and 94% amino acid sequence [11], they are serologically indistinguishable [12] and are both diagnosed as Powassan virus.

**Figure 2:**
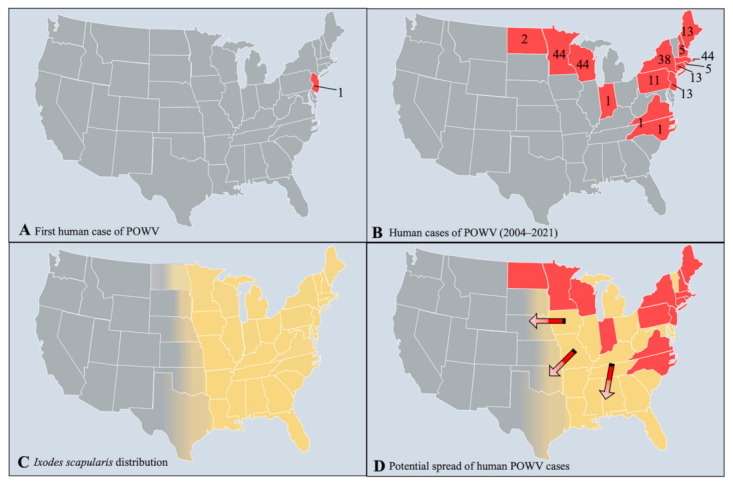
The distribution of Powassan virus cases and *I. scapularis.* (**A**) The first initial case of POWV in New Jersey, US in 1970. (**B**) Human cases of POWV from 2004–2021 (as of 29 October 2021), reported by the CDC and ArboNet. (**C**) The distribution of *I. scapularis* based on data from the CDC. (**D**) The potential range expansion of POWV cases based on the distribution of *I. scapularis.* Red states indicate current POWV presence, yellow states designate *I. scapularis* distribution, and arrows show the potential direction of spread for POWV.

**Figure 3:**
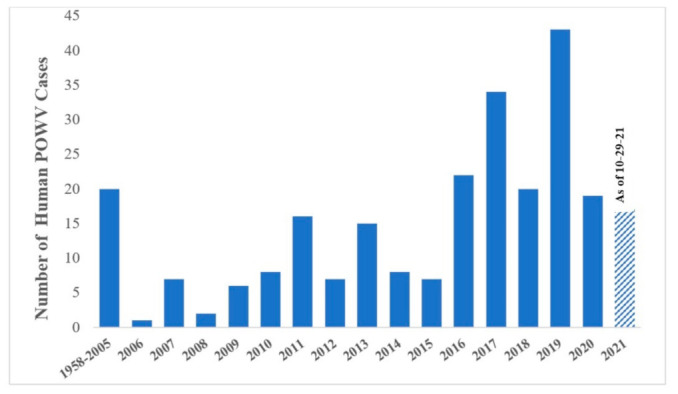
The number of human POWV cases reported from 1958–2021. From 1958–2005, only 20 cases were reported. From 2006–2020, an average of 14 cases a year were recorded [13,16]. Data for 2021 is represented up to 29 October 2021.

Isolates from both lineages are found in the US, Canada, and Far Eastern Russia [13,14], and fourteen states in the US now report POWV cases including Connecticut, Indiana, Maine, Massachusetts, Minnesota, New Hampshire, New Jersey, New York, North Carolina, North Dakota, Pennsylvania, Rhode Island, Virginia, and Wisconsin (Figure 2) [15,16]. Clinical cases of POWV are defined by the CDC as having a fever, central and peripheral nervous system dysfunction, and the absence of a more likely clinical explanation [17]. Human cases are restricted to the distribution range of the tick vector, namely in the Northeast and Upper Midwest states, and they are rising in incidence (Figure 3). Prior to 2006, only 20 cases were reported to the CDC [13]. However, between 2010 and 2019 alone, 181 cases were reported [15]. This increase is likely due to increased surveillance and reporting (POWV neuroinvasive and non-neuroinvasive diseases were added to the list of nationally notifiable diseases in 2001 and 2004, respectively), improved diagnosis, and/or increased prevalence [13,18]. With POWV disease being a rare tick-borne disease that can also have non-specific symptoms, it is likely that cases are underestimated and/or misdiagnosed, and the true extent of the case geographic distribution cannot be determined [19]. 

Unlike *B. burgdorferi*, POWV can be deadly, with a 3–35.7% case fatality rate and long-term neurological damage in 50% of survivors [19,20]. Importantly, both strains of the virus can cause fatal neurological disease [20,21], and common symptoms can include encephalitis, meningitis, aseptic meningitis, febrile illness, lethargy, weakness, confusion, headaches, and vomiting [13,22]. All age groups can be affected, although a shift from younger individuals to older individuals over the decades has been seen [23]. From 2006 to 2016, ages of cases ranged from 3 to 87 with a mean of 62, and most deaths occurred in people over 50 years of age [13]. 

Since there are no FDA approved vaccines available for POWV and the incidence of this virus is increasing, it is pertinent to highlight what is known about the vectors, reservoir (virus-amplifying) hosts, transmission cycles, and, consequently, virus spillover to humans.

## 2. Vector and Host Associations

After the first fatal case of POWV, targeted field investigations looked towards ticks as the vector since POWV is serologically related to Russian spring-summer encephalitis virus, a known tick-borne virus [24]. Unsurprisingly, researchers did discover that Ixodid ticks were responsible. POWV-positive *Ixodes marxi* (squirrel tick) were collected from a red squirrel (*Tamiasciurus hudsonicus*), and the virus was isolated from the blood of another squirrel, suggesting a potential enzootic cycle for POWV (Figure 1) [25]. Continued surveillance found *I. cookei* (groundhog tick) harvested from groundhogs (*Marmota monax*) infected with the virus as well as groundhogs with neutralizing antibodies and viral presence in blood (Figure 1) [26]. Additional studies implicated *I. cookei* as the vector for POWV (strain not specified) [27,28,29]. Furthermore, in 1997, *I. scapularis* was implicated as the vector for DTV (Figure 1) [10], supported by other studies [30,31,32]. Unfortunately, prior to the discovery of DTV, most earlier studies of POWV did not differentiate between both strains. However, Whitney and Jamnback (1965) detected POWV-LB in *I. cookei* [33]. Moreover, while a retrospective study found *Dermacentor andersoni* ticks collected in 1952 with POWV isolates, which was later identified as DTV (Figure 1) [19,34], the overall transmission potential of these ticks is unknown and understudied. *D. andersoni* ticks are found in the western US, in areas surrounding the Rocky Mountains [35]. With the lack of human POWV cases in that region, little evidence to implicate this vector, and field collected *D. andersoni* testing negative for POWV [31], it is unlikely that these ticks play a significant role in POWV maintenance at this time. Through collective research, it is accepted that the main vector of POWV-LB is *I. cookei* and DTV is *I. scapularis*, though little research exists on other tick species. 

A number of field-collected vertebrates were found with neutralizing antibodies for POWV, indicating prior infection, including striped skunks, short-tailed and long-tailed weasels, racoons, porcupines, red squirrels, gray squirrels, groundhogs, opossums, some birds, and chipmunks [18,25,27,29,36,37]. However, the exact role in host competency and virus amplification is unclear. 

While the enzootic cycles sustaining POWV-LB are purported to be between *I. cookei* and groundhogs and mustelids, and, to a lesser extent, *I. marxi* and squirrels, conclusive evidence for a wildlife reservoir of DTV is lacking. It is known that larval and nymphal *I. scapularis* frequently parasitize white-footed mice (*Peromyscus leucopus*) in the US, and this vector-host relationship robustly sustains *B. burgdorferi* in nature. It is often assumed in the literature that *P. leucopus* is also the reservoir for DTV. One study found seropositive wild-caught *P. leucopus* and DTV-infected *I. scapularis* in the same site, but the seroreactivity could have been from either POWV-LB or DTV [30]. Two *Peromyscus* spp. also had DTV antibodies in New Mexico, but this could not be confirmed in the absence of any vectors, isolates, or sequence data [38]. Moreover, while *I. scapularis*-infested *P. leucopus* were found to frequently enter groundhog burrows at night, fostering the ability for vectors and potential reservoirs to converge, no testing was done on any vertebrates or ticks to determine infection status [39]. Furthermore, *I. scapularis* collected from *P. leucopus* tested negative for DTV [37]. To date, DTV has yet to be isolated from wild-caught *Peromyscus* spp. to effectively implicate this host as a reservoir. 

It is possible that potential reservoirs for DTV are small burrowing rodents such as voles because, unlike *B. burgdorferi*, DTV appears focally, even though it is transmitted by the same vector species. Because DTV is understudied, a lot of what is known is extrapolated from our knowledge about tick-borne encephalitis virus (TBEV), a flavivirus closely related to POWV that circulates in Europe. Specifically, POWV and DTV are members of the TBEV complex. In Europe, there is strong evidence that voles (*Myodes* spp.) are the natural reservoirs for TBEV. TBEV was isolated from the brain of voles in Slovakia [40] and from the spleen, lung, and kidney in wild voles in the Czech Republic [41]. In Finland, voles tested positive for TBEV with RNA detected in the brain [42]. Additional studies demonstrated proportions of the *Myodes* population displaying seropositivity for TBEV [43,44]. Since voles appear to remain asymptomatic albeit having high viral load, this can contribute to sustaining the virus in nature [45]. There is not much evidence for this happening with DTV in the US yet, as no voles were found seropositive in the Northeast [30]. However, more research needs to be carried out to implicate this host. 

## 3. Transmission Dynamics

**Figure 4:**
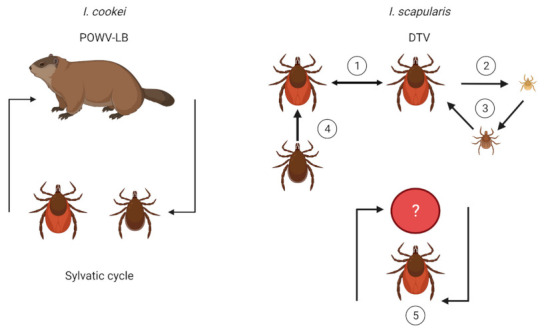
Transmission dynamics of POWV in *I. cookei* and *I. scapularis.* The maintenance of POWV-LB is purported to be between groundhogs and *I. cookei*; however additional mechanisms of transmission may exist, similar to DTV. *I. scapularis* may maintain DTV in nature through (1) cofeeding on a host, irrespective of host viremia, (2) vertical transmission, (3) transstadial transmission, (4) venereal transmission, and (5) a sylvatic cycle with an unknown reservoir. Illustrations were created using Biorender (biorender.com, accessed on 30 September 2021).

As with other tick-borne viruses, multiple modes of transmission exist for POWV (Figure 4). Since both lineages are so similar, it can be expected that transmission mechanisms are likely the same. However, less research has been performed on *I. cookei* and POWV-LB since humans have more exposure to *I. scapularis* and DTV. With the assumption that both lineages have similar transmission mechanisms, this section will discuss POWV-LB and DTV concurrently as POWV. Concerningly, a unique feature of POWV is that it can transmit to the mammalian host in as little as 15 min of tick attachment [46], compared with *B. burgdorferi* which transmits between 24–48 h [47]. While this transmission study occurred in a mouse model, human cases have been found to occur with tick attachment in as little as 3–6 h [48]. The quick dissemination from tick to host may occur since POWV virus is already present in the salivary glands prior to the acquisition of the next blood meal [46], as opposed to the *B. burgdorferi* spirochete which is housed in the midgut before migrating to the salivary glands [49]. Furthermore, it has been shown that tick saliva enhances POWV transmission (Figure 1) [50]. Due to the rapid transmission rate and lack of grace period for tick removal, POWV has a high potential for causing disease in humans, exacerbated by the fact that nymphs can transmit the virus and are less detectable because of their small size. POWV also transmits transtadially [46,51], and there is minor evidence of vertical transmission to offspring [51]. However, more confirmatory research is needed. Venereal transmission may also facilitate transmission if the infected saliva from a male coats the spermatophore during transfer to an uninfected female [51]. Furthermore, some evidence has been shown for the transmission of POWV through infected milk [52], and this has also been demonstrated for TBEV transmission [53,54]. 

One hypothesis for the focality of and low prevalence of POWV is that the virus is sustained predominantly through cofeeding transmission where aggregating uninfected and infected ticks feed in close proximity and transmit the virus to one another. Cofeeding transmission can still occur if a host is non-viremic [55], and evidence for non-viremic infection through aggregated cofeeding has been shown for TBEV and other tick-borne viruses [56,57,58,59].

Ticks are capable of being infected with multiple pathogens simultaneously, and previous research has demonstrated that pathogens may behave synergistically, facilitating transmission. For example, the presence of both *B. burgdorferi* and *Babesia microti* will suppress the host’s immune response and can worsen clinical outcomes [60,61], and *B. burgdorferi* may also increase the incidence of *B. microti* in ticks, aiding in its geographic expansion [62]. Mosquito flaviviruses like dengue and Zika virus will cross-react and have an antibody-dependent enhancement response, where dengue antibodies will enhance cell infection by Zika virus [63,64,65]. However, the extent to which coinfections help facilitate the transmission of POWV in nature is currently unknown, and little information is available on the coinfections of tick-borne viruses. Nonetheless, the number of coinfected ticks in the US is staggering, namely in the Northeast US where *B. burgdorferi* is widespread [66], and more research needs to be carried out to elucidate the transmission potential of coinfected ticks.

Transmission potential is influenced by many factors, but it largely begins with host preference. *I. scapularis* is a generalist and will feed on a wide array of vertebrates. It is likely that humans will be incidental hosts, as seen with the growing number of Lyme disease cases [6]. Previously, it was thought that *I. cookei* was predominantly nidicolous, remaining in burrows and being highly host-specific [67]. However, questing *I. cookei* have been collected through public passive surveillance in New York (www.nyticks.org, accessed on 21 October 2021), Maine [68], and Pennsylvania [69]. While, *I. cookei* does have the potential to cause virus spillover to humans, *I. scapularis* is likely more of a threat due to its wider distribution range and more frequent human encounters [68,69]. However, the true potential for these vectors has yet to be investigated.

## 4. Spatial, Temporal, Habitat, and Meteorological Associations

Since POWV is maintained in nature by Ixodid ticks, the presence of POWV depends on the geographic location where the vectors are found and suitable environmental factors that support the vector population. With transmission cycles primarily sustained by *I. cookei* and *I. scapularis*, discussions of environmental associations will focus on these two species. However, compared to *I. scapularis*, research on *I. cookei* is sparse since it historically is encountered less by the public. *I. scapularis* and *I. cookei* are found throughout the eastern half of the US, with concentrations in the Northeast, Upper Midwest, and Great Lakes regions, and the distribution range is expanding [69,70,71]. *I. scapularis* has a larger distribution range compared to *I. cookei*, which may assist its ability to vector POWV more effectively to a larger population.

**Figure 5:**
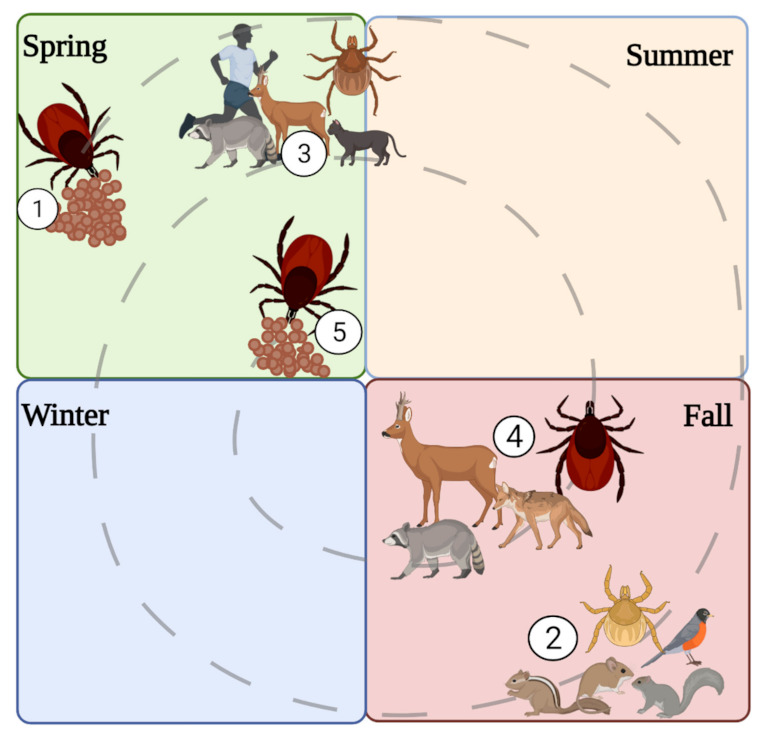
Life cycle of *I. scapularis*. (1) Female tick lays eggs in the spring. (2) The larvae hatch in the fall and feed on small mammals and birds. (3) The larvae overwinter and molt into nymphs. The nymph’s quest on medium to large mammals. (4) The nymphs molt into adults, and the adults quest on large mammals. (5) The adults overwinter, and the females lay eggs in spring and then die. Illustrations were created using Biorender (biorender.com, accessed on 30 September 2021).

The seasonality of tick life stages influences transmission potential since, during periods of host-seeking activity, ticks actively bite and pass the virus to hosts. *I. scapularis* has a two-year life cycle where adult ticks quest in spring and late fall (March–April; October–November), nymphs in early spring (June–July), and larvae in early fall (July–August; Figure 5) [72]. Comparatively, *I. cookei* activity occurred all year, with nymph and adult activity peaking in July while larvae activity showed fluctuations from March through December [69]. In the interim, the tick is dormant while digesting and molting into the next life stage. Consequently, human cases of POWV disease coincide with tick activity (i.e., late spring, early summer, and mid-fall) [73].

Ixodid ticks spend most of their life exposed to the environment, and thus, their survival depends on optimal ecological conditions. Suitable vegetation is necessary for tick survival, and vegetation is impacted by soil type which influences water drainage efficiency. Consequently, sandy soils support dry and dry/mesic forests which grow oak trees, and this environment positively correlated to the *I. scapularis* population [74], likely due to the fact that immature stages require moisture retention in the leaf litter duff layer to avoid desiccation and ideal hosts, such as the white-footed mouse and other small rodents, are prominent in oak habitats. Likewise, *I. cookei* is found in temperate and broadleaf forests, common in the eastern US [75]. Additionally, immature *I. scapularis* feed on white-footed mice and other small rodents, and these populations are impacted by food availability, namely fruitful mast years with abundant seeds [76]. Thus, the increase in host food sources will effectively support the host population, and in turn, sustain the vector population.

Meteorological variables, like temperature, humidity, and rainfall, affect moisture content in microhabitats, influencing tick reproduction rate, desiccation status, and host food availability. *I. scapularis* can typically be found in higher humid environments where they will be less vulnerable to desiccation. For instance, they were found questing more often during the morning where humidity was higher and temperature was lower [77], and hot, dry weather decreased the overall abundance of questing *I. scapularis* nymphs [78]. Certain habitats, such as Japanese barberry (*Berberis thunbergii*), retain humidity which is favorable for *I. scapularis* [79]. Furthermore, host food sources, such as those produced during mast years, are impacted by temperature, precipitation, and frost conditions which, in turn, affect the density of the rodent population [76]. While meteorological conditions impact the presence of ticks, conditions that are favorable for the external incubation period for POWV are currently unknown.

## 5. Strain Variation and Stability

Multiple POWV strains exist in nature, but what causes certain strains to present in specific foci remains speculative. It has become evident that POWV is highly focal and stable [80]. In Connecticut, two distinct subclades were found only 40 km apart [81]. Likewise, a 5 km^2^ TBEV focus in Germany revealed two circulating clades of virus that occupied different habitats (forest-meadow ecotone or forest). Over a decade, one clade showed more annual stability while the other appeared more sporadically. Moreover, nymphal counts varied annually. However, the minimum infection rate remained stable [82]. Similarly, in a Wisconsin focus, the proportion of infected ticks remained the same, even though tick density increased over a decade [31]. It is possible that certain strains may be found in more exclusive habitats, possibly driven by specific host populations in those habitats or other ecological variables not yet examined. More research should be conducted to elucidate the ecological mechanisms sustaining specific POWV strains in nature.

## 6. Climate Change and Anthropogenic Influence

The changing climate and landscape are impacting the presence of tick and host species, and, subsequently, are affecting the presence of tick-borne pathogens. Global climate change is leading towards rising annual temperatures, caused largely in part by the excess release of carbon dioxide and methane [83]. Resulting extreme weather events, such as hurricanes, droughts, and flooding, target global regions differently, and this can impact the growing distribution potential of vector populations [84,85]. In the US, ecological and climate modeling has predicted a growing distribution of *I. cookei* northward into Canada and a decreasing presence southward in the US, while *I. scapularis* is predicted to expand northward and westward [71], exposing new areas to tick-borne pathogens. Similarly, the white-footed mouse is expected to also expand northward and colonize new areas as temperature rises and winter length shortens [86], and this northward movement may be supported by earlier oak flowering which is correlated to rising spring temperatures earlier in the year [87]. It is possible that this effect may support other rodent species and pathogen reservoirs. Also, with increasing temperatures and shorter winters, groundhogs may exhibit less time in hibernation and more time actively outside of burrows, reproducing [88]. However, whether this has an impact on *I. cookei* population density or POWV prevalence is unknown.

In the early 1800s, Japanese barberry was introduced from Japan to the US, and it became a popular ornamental plant that has since invaded much of the Northeast and Upper Midwest [89]. Found in wooded environments, Japanese barberry is ideal for *I. scapularis* populations [79,90], it coincides with *I. scapularis* distribution [35,89], and it both grows densely and produces thorns, deterring predators while creating a safe haven for rodents. Moreover, the berries may also provide a food source that supports the rodent population. Consequently, the role of Japanese barberry in maintaining *I. scapularis*, hosts, and virus should be examined.

With the encroachment of humans into wildlife habitats, large tracts of land are becoming fragmented for urbanization and agriculture. Habitat fragmentation inadvertently leads to the loss of biodiversity and the concentration of adaptable hosts into pockets of natural vegetation. Specifically, white-footed mice and voles (and perhaps other generalist small mammals) are successful at adapting to the fragmented landscape [91,92], which could contribute to successful foci of POWV. Conversely, larger habitat patch sizes support more biodiversity and offer a protective “dilution effect” for ticks and tick-borne pathogens, decreasing disease prevalence [93]. With decreasing forest patch sizes, we may expect to see increased densities of *I. scapularis* [94], though fragmentation effects on POWV are unknown since the virus presents focally. Moreover, its effect on *I. cookei* is undetermined since the tick may effectively be sustained in burrows. Furthermore, without knowing the reservoir for DTV, we cannot be sure how habitat loss directly impacts DTV prevalence.

While humans are contributing to global climate change and habitat reduction, increasing tick-borne disease prevalence, there are a number of ways that people can physically reduce tick densities and personal tick-borne pathogen acquisition, including POWV. Personal preventative measures for reducing pathogen attainment include frequently checking for ticks when outdoors, staying in the center of maintained trails, wearing EPA-approved repellents, wearing light-colored clothing, and tucking one’s pants into their socks [95]. Prevention methods at home include cultivating a tick-free yard space by reducing leaf litter on the ground, removing weeds and bramble that may attract small rodents, creating a mulch barrier between the woods and the yard, and allowing ample sunlight to dry out the yard (reducing moisture for ticks) by regularly mowing and landscaping properly [95]. Host-targeted acaricides also are available to treat rodents and deer through passive topical applications [95]. By reducing hosts, treating hosts for ticks, landscaping to reduce tick abundance, and practicing personal prevention methods, tick-borne pathogen spillover to humans can be reduced. However, these methods are on the individual level, and long-term solutions require global participation.

## 7. Conclusions

Tick-borne pathogens are rising in incidence, and there are currently no vaccines in the US to curb the spread of these pathogens to humans. POWV is a rare and deadly virus causing a disease that is growing in incidence with two major tick species supporting the virus in nature: *I. scapularis* and *I. cookei.* Both ticks will bite and transmit the virus to humans, and the distribution range of these ticks is growing. The relationship between pathogen, host, vector, and environment is complex and highly interdependent. Research is ongoing to elucidate what sustains POWV in localized areas as well as environmental variables that may facilitate the maintenance and spread of vector and pathogen. The best practices for reducing POWV disease include practicing personal tick prevention methods to decrease tick bites.

## Data Availability

Not applicable.

## References

[1] WHO WHO Coronavirus (COVID-19) Dashboard. https://covid19.who.int/.

[2] Sonenshine D.E. (2018). Range expansion of tick disease vectors in north america: Implications for spread of tick-borne disease. Int. J. Environ. Res. Public Health.

[3] Diuk-Wasser M.A., VanAcker M.C., Fernandez M.P. (2021). Impact of Land Use Changes and Habitat Fragmentation on the Eco-epidemiology of Tick-Borne Diseases. J. Med. Entomol..

[4] Gould E.A., Higgs S. (2009). Impact of climate change and other factors on emerging arbovirus diseases. Trans. R. Soc. Trop. Med. Hyg..

[5] Paddock C., Lane R., Staples J., Labruna M. (2016). Changing paradigms for tick-borne diseases in the Americas. Global Health Impacts of Vector-Borne Diseases.

[6] CDC (2021). How Many People Get Lyme Disease?. https://www.cdc.gov/lyme/stats/humancases.html.

[7] Paules C., Marston H., Bloom M., Fauci A. (2018). Tickborne Diseases—Confronting a Growing Threat. N. Engl. J. Med..

[8] McLean D.M., Donohue W.L. (1959). Powassan virus: Isolation of virus from a fatal case of encephalitis. Can. Med. Assoc. J..

[9] Goldfield M., Austin S.M., Black H.C., Taylor B.F., Altman R. (1973). A non-fatal human case of Powassan virus encephalitis. Am. J. Trop. Med. Hyg..

[10] Telford S.R., Armstrong P.M., Katavolos P., Foppa I., Garcia A.S.O., Wilson M.L., Spielman A. (1997). A new tick-borne encephalitis-like virus infecting New England deer ticks, Ixodes dammini. Emerg. Infect. Dis..

[11] Kuno G., Artsob H., Karabatsos N., Tsuchiya K.R., Chang G.J.J. (2001). Genomic sequencing of deer tick virus and phylogeny of Powassan-related viruses of North America. Am. J. Trop. Med. Hyg..

[12] Beasley D.W.C., Suderman M.T., Holbrook M.R., Barrett A.D.T. (2001). Nucleotide sequencing and serological evidence that the recently recognized deer tick virus is a genotype of Powassan virus. Virus Res..

[13] Krow-Lucal E.R., Lindsey N.P., Fischer M., Hills S.L. (2018). Powassan virus disease in the United States, 2006–2016. Vector-Borne Zoonotic Dis..

[14] Leonova G.N., Kondratov I.G., Ternovoi V.A., Romanova E.V., Protopopova E.V., Chausov E.V., Pavlenko E.V., Ryabchikova E.I., Belikov S.I., Loktev V.B. (2009). Characterization of Powassan viruses from Far Eastern Russia. Arch. Virol..

[15] CDC (2019). Powassan Virus. Centers for Disease Control and Prevention. https://www.cdc.gov/powassan/statistics.html.

[16] CDC (2021). Powassan Virus. https://wwwn.cdc.gov/arbonet/Maps/ADB_Diseases_Map/index.html.

[17] CDC (2021). Arboviral Diseases, Neuroinvasive and Non-Neuroinvasive 2015 Case Definition. https://ndc.services.cdc.gov/case-definitions/arboviral-diseases-neuroinvasive-and-non-neuroinvasive-2015/.

[18] Hinten S.R., Beckett G.A., Gensheimer K.F., Pritchard E., Courtney T.M., Sears S.D., Woytowicz J.M., Preston D.G., Smith R.P., Rand P.W. (2008). Increased recognition of powassan encephalitis in the United States, 1999–2005. Vector-Borne Zoonotic Dis..

[19] Corrin T., Greig J., Harding S., Young I., Mascarenhas M., Waddell L.A. (2018). Powassan virus, a scoping review of the global evidence. Zoonoses Public Health.

[20] Ebel G.D. (2010). Update on Powassan virus: Emergence of a North American tick-borne flavivirus. Annu. Rev. Entomol..

[21] Hermance M.E., Thangamani S. (2017). Powassan virus: An emerging arbovirus of public health concern in North America. Vector-Borne Zoonotic Dis..

[22] El Khoury M.Y., Camargo J.F., White J.L., Backenson B.P., Dupuis A.P., Escuyer K.L., Kramer L., George K.S., Chatterjee D., Prusinski M. (2013). Potential Role of Deer Tick Virus in Powassan Encephalitis Cases in Lyme Disease–endemic Areas of New York, USA. Emerg. Infect. Dis..

[23] Campbell O., Krause P.J. (2020). The emergence of human Powassan virus infection in North America. Ticks Tick. Borne. Dis..

[24] Mclean D.M., Walker S.J., Macpherson L.W., Scholten T.H., Ronald K., Wyllie J.C., Mcqueen E.J. (1961). Powassan Virus: Investigations of possible natural cycles of infection. J. Infect. Dis..

[25] McLean D.M., Larke R.P. (1963). Powassan and Silverwater viruses: Ecology of two Ontario arboviruses. Can. Med. Assoc. J..

[26] Mclean D.M., Best J.M., Sc B., Ass J. (1964). Powassan virus: Summer infection cycle, 1964. Can. Med. Assoc. J..

[27] Main A.J., Carey A.B., Downs W.G., Haven N. (1979). Powassan virus in Ixodes cookei and mustelidae in New England. J. Wildl. Dis..

[28] McLean D.M., Cobb C., Gooderham S.E., Smart C.A., Wilson A.G., Wilson W.E. (1967). Powassan virus: Persistence of virus activity during 1966. Can. Med. Assoc. J..

[29] Mclean D.M., de Vos A., Quantz J. (1964). Powassan virus: Field investigations of 1963. Am. J. Trop. Med. Hyg..

[30] Ebel G.D., Campbell E.N., Goethert H.K., Spielman A., Telford S.R. (2000). Enzootic transmission of deer tick virus in new England and Wisconsin sites. Am. J. Trop. Med. Hyg..

[31] Brackney D.E., Nofchissey R.A., Fitzpatrick K.A., Brown I.K., Ebel G.D. (2008). Short report: Stable prevalence of Powassan virus in Ixodes scapularis in a Northern Wisconsin focus. Am. J. Trop. Med. Hyg..

[32] Tokarz R., Tagliafierro T., Cucura D.M., Rochlin I., Sameroff S., Lipkin W.I. (2017). Detection of Anaplasma phagocytophilum, Babesia microti, Borrelia burgdorferi, Borrelia miyamotoi, and Powassan Virus in Ticks by a Multiplex Real-Time Reverse Transcription-PCR Assay. mSphere.

[33] Whitney E., Jamnback H. (1965). The first isolations of Powassan virus in New York State. Proc. Soc. Exp. Biol. Med..

[34] Thomas L.A., Kennedy R.C., Eklund C.M. (1960). Isolation of a virus closely related to Powassan virus from Dermacentor andersoni collected along North Cache la Poudre River, Colorado. Proc. Soc. Exp. Biol. Med..

[35] CDC (2019). Geographic Distribution of Ticks that Bite Humans. https://www.cdc.gov/ticks/geographic_distribution.html.

[36] Johnson H.N. (1987). Isolation of Powassan virus from a spotted skunk in California. J. Wildl. Dis..

[37] Dupuis A.P., Peters R.J., Prusinski M.A., Falco R.C., Ostfeld R.S., Kramer L.D. (2013). Isolation of deer tick virus (Powassan virus, lineage II) from Ixodes scapularis and detection of antibody in vertebrate hosts sampled in the Hudson Valley, New York State. Parasites Vectors.

[38] Deardorff E.R., Nofchissey R.A., Cook J.A., Hope A.G., Tsvetkova A., Talbot S.L., Ebel G.D. (2013). Powassan Virus in mammals, Alaska and New Mexico, USA, and Russia, 2004–2007. Emerg. Infect. Dis..

[39] Sprague A. (2010). Down the Woodchuck Hole: Investigating the Potential for Peromyscus Leucopus to Act as a Mixing Vessel for the Powassan Virus and Deer Tick Virus Disease Systems (Issue Usgs).

[40] Frey S., Essbauer S., Zöller G., Klempa B., Dobler G., Pfeffer M. (2014). Full genome sequences and preliminary molecular characterization of three tick-borne encephalitis virus strains isolated from ticks and a bank vole in Slovak Republic. Virus Genes.

[41] Weidmann M., Schmidt P., Hufert F.T., Krivanec K., Meyer H. (2006). Tick-borne encephalitis virus in Clethrionomys glareolus in the Czech Republic. Vector-Borne Zoonotic Dis..

[42] Tonteri E., Kipar A., Voutilainen L., Vene S., Vaheri A., Vapalahti O., Lundkvist Å. (2013). The three subtypes of tick-borne encephalitis virus induce encephalitis in a natural host, the bank vole (Myodes glareolus). PLoS ONE.

[43] Burri C., Korva M., Bastic V., Knap N., Avšič-Županc T., Gern L. (2012). Serological Evidence of Tick-Borne Encephalitis Virus Infection in Rodents Captured at Four Sites in Switzerland. J. Med. Entomol..

[44] Zöldi V., Papp T., Reiczigel J., Egyed L. (2015). Bank voles show high seropositivity rates in a natural TBEV focus in Hungary. Infect. Dis..

[45] Mlera L., Bloom M.E. (2018). The role of mammalian reservoir hosts in tick-borne flavivirus biology. Front. Cell. Infect. Microbiol..

[46] Ebel G.D., Kramer L.D. (2004). Short report: Duration of tick attachment required for transmission of powassan virus by deer ticks. Am. J. Trop. Med. Hyg..

[47] Hermance M.E., Thangamani S. (2018). Tick^−^Virus^−^Host Interactions at the Cutaneous Interface: The Nidus of Flavivirus Transmission. Viruses.

[48] Feder H.M., Telford S., Goethert H.K., Wormser G.P. (2020). Powassan virus encephalitis following brief attachment of Connecticut deer ticks. Clin. Infect. Dis..

[49] Spielman A., Ribeiro J.M.C., Mather T.N., Piesman J. (1987). Dissemination and salivary delivery of Lyme disease spirochetes in vector ticks (Acari: Ixodidae). J. Med. Entomol..

[50] Hermance M.E., Thangamani S. (2015). Tick saliva enhances Powassan virus transmission to the host, influencing its dissemination and the course of disease. J. Virol..

[51] Costero A., Grayson M.A. (1996). Experimental transmission of Powassan virus (Flaviviridae) by Ixodes scapularis ticks (Acari:Ixodidae). Am. J. Trop. Med. Hyg..

[52] Woodall J.P., Roz A. (1977). Experimental milk-borne transmission of Powassan virus in the goat. Am. J. Trop. Med. Hyg..

[53] Dorko E., Hockicko J., Rimárová K., Bušová A., Popaďák P., Popaďáková J., Schréter I. (2018). Milk outbreaks of tick-borne encephalitis in Slovakia, 2012-2016. Cent. Eur. J. Public Health.

[54] Cisak E., Wójcik-Fatla A., Zając V., Sroka J., Buczek A., Dutkiewicz J. (2010). Prevalence of tick-borne encephalitis virus (TBEV) in samples of raw milk taken randomly from cows, goats and sheep in eastern Poland. Ann. Agric. Environ. Med..

[55] Jones L.D., Davies C.R., Steele G.M., Nuttall P.A. (1987). A novel mode of arbovirus transmission involving a nonviremic host. Science.

[56] Havlíková S., Licková M., Klempa B. (2013). Non-viraemic transmission of tick-borne viruses. Acta Virol..

[57] Labuda M., Danielova V., Jones L.D., Nuttall P.A. (1993). Amplification of tick-borne encephalitis virus infection during co-feeding of ticks. Med. Vet. Entomol..

[58] Labuda M., Jones L.D., Williams T., Danielova V., Nuttall P.A. (1993). Efficient transmission of tick-borne encephalitis virus between cofeeding ticks. J. Med. Entomol..

[59] Labuda M., Kozuch O., Zuffová E., Elecková E., Hails R.S., Nuttall P.A. (1997). Tick-borne encephalitis virus transmission between ticks cofeeding on specific immune natural rodent hosts. Virology.

[60] Martínez-Balzano C., Hess M., Malhotra A., Lenox R. (2015). Severe babesiosis and Borrelia burgdorferi co-infection. QJM An. Int. J. Med..

[61] Vinasco J., Braga W., Zegarra-Moro O., Moro M.H. (2007). Cellular immune responses in a murine model of Borrelia burgdorferi and Babesia microti coinfection. J. Immunol..

[62] Dunn J.M., Krause P.J., Davis S., Vannier E.G., Fitzpatrick M.C., Rollend L., Belperron A.A., States S.L., Stacey A., Bockenstedt L.K. (2015). Borrelia burgdorferi promotes the establishment of Babesia microti in the Northeastern United States. PLoS ONE.

[63] Charles A.S., Christofferson R.C. (2016). Utility of a dengue-derived monoclonal antibody to enhance Zika infection in vitro. PLoS Curr..

[64] Durbin A.P. (2016). Dengue antibody and Zika: Friend or foe?. Trends Immunol..

[65] Paul L.M., Carlin E.R., Jenkins M.M., Tan A.L., Barcellona C.M., Nicholson C.O., Michael S.F., Isern S. (2016). Dengue virus antibodies enhance Zika virus infection. Clin. Transl. Immunol..

[66] Lehane A., Maes S.E., Graham C.B., Jones E., Delorey M., Eisen R.J. (2021). Prevalence of single and coinfections of human pathogens in Ixodes ticks from five geographical regions in the United States, 2013–2019. Ticks Tick-Borne Dis..

[67] Ko R.C. (1972). Biology of Ixodes cookei Packard (Ixodidae) of groundhogs (Marmota monax Erxleben). Can. J. Zool..

[68] Rand P.W., Lacombe E.H., Dearborn R., Cahill B., Elias S., Lubelczyk C.B., Beckett G.A., Smith R.P. (2007). Passive surveillance in Maine, an area emergent for tick-borne diseases. J. Med. Entomol..

[69] Pak D., Jacobs S.B., Sakamoto J.M. (2019). A 117-year retrospective analysis of Pennsylvania tick community dynamics. Parasites Vectors.

[70] Eisen R.J., Eisen L., Beard C.B. (2016). County-scale distribution of Ixodes scapularis and Ixodes pacificus (Acari: Ixodidae) in the continental United States. J. Med. Entomol..

[71] Alkishe A., Raghavan R.K., Peterson A.T. (2021). Likely geographic distributional shifts among medically important tick species and tick-associated diseases under climate change in North America: A review. Insects.

[72] Simmons T.W., Shea J., Myers-Claypole M.A., Kruise R., Hutchinson M.L. (2015). Seasonal activity, density, and collection efficiency of the blacklegged tick (Ixodes scapularis) (Acari: Ixodidae) in Mid-Western Pennsylvania. J. Med. Entomol..

[73] CDC (2020). How Ticks Spread Disease. https://www.cdc.gov/ticks/life_cycle_and_hosts.html.

[74] Guerra M., Walker E., Jones C., Paskewitz S., Roberto Cortinas M., Ashley Stancil L.B., Bobo M., Kitron U. (2002). Predicting the risk of Lyme disease: Habitat suitability for Ixodes scapularis in the north central United States. Emerg. Infect. Dis..

[75] Guglielmone A.A., Robbins R.G., Apanaskevich D.A., Petney T.N., Estrada-Peña A., Horak I.G. (2014). The Hard Ticks of the World.

[76] Clotfelter E.D., Pedersen A.B., Cranford J.A., Ram N., Snajdr E.A., Nolan V., Ketterson E.D. (2007). Acorn mast drives long-term dynamics of rodent and songbird populations. Oecologia.

[77] Schulze T.L., Jordan R.A., Hung R.W. (2001). Effects of selected meteorological factors on diurnal questing of Ixodes scapularis and Amblyomma americanum (Acari: Ixodidae). J. Med. Entomol..

[78] Burtis J.C., Sullivan P., Levi T., Oggenfuss K., Fahey T.J., Ostfeld R.S. (2016). The impact of temperature and precipitation on blacklegged tick activity and Lyme disease incidence in endemic and emerging regions. Parasites Vectors.

[79] Elias S.P., Lubelczyk C.B., Rand P.W., Lacombe E.H., Holman M.S., Smith Jr R.P. (2006). Deer browse resistant exotic-invasive understory: An indicator of elevated human risk of exposure to Ixodes scapularis (Acari: Ixodidae) in southern coastal Maine woodlands. J. Med. Entomol..

[80] Pesko K.N., Torres-Perez F., Hjelle B.L., Ebel G.D. (2010). Molecular epidemiology of Powassan virus in North America. J. Gen. Virol..

[81] Anderson J.F., Armstrong P.M. (2012). Prevalence and genetic characterization of Powassan virus strains infecting Ixodes scapularis in Connecticut. Am. J. Trop. Med. Hyg..

[82] Borde J.P., Kaier K., Hehn P., Matzarakis A., Frey S., Bestehorn M., Dobler G., Chitimia-Dobler L. (2021). The complex interplay of climate, TBEV vector dynamics and TBEV infection rates in ticks—Monitoring a natural TBEV focus in Germany, 2009–2018. PLoS ONE.

[83] Jorgenson A.K. (2006). Global warming and the neglected greenhouse gas: A cross-national study of the social causes of methane emissions intensity, 1995. Soc. Forces.

[84] Ogden N.H., Ben Beard C., Ginsberg H.S., Tsao J.I. (2021). Possible effects of climate change on Ixodid ticks and the pathogens they transmit: Predictions and observations. J. Med. Entomol..

[85] Ebi K.L., Vanos J., Baldwin J.W., Bell J.E., Hondula D.M., Errett N.A., Hayes K., Reid C.E., Saha S., Spector J. (2020). Extreme weather and climate change: Population health and health system implications. Annu. Rev. Public Health.

[86] Roy-Dufresne E., Logan T., Simon J.A., Chmura G.L., Millien V. (2013). Poleward expansion of the white-footed mouse (*Peromyscus leucopus*) under climate change: Implications for the spread of lyme disease. PLoS ONE.

[87] Caignard T., Kremer A., Firmat C., Nicolas M., Venner S., Delzon S. (2017). Increasing spring temperatures favor oak seed production in temperate areas. Sci. Rep..

[88] Zervanos S.M., Maher C.R., Waldvogel J.A., Florant G.L. (2010). Latitudinal differences in the hibernation characteristics of woodchucks (Marmota monax). Physiol. Biochem. Zool..

[89] EDDMapS (2021). Japanese Barberry. https://www.eddmaps.org/distribution/uscounty.cfm?sub=3010.

[90] Williams S.C., Ward J.S. (2010). Effects of Japanese barberry (Ranunculales: Berberidaceae) removal and resulting microclimatic changes on Ixodes scapularis (Acari: Ixodidae) abundances in Connecticut, USA. Environ. Entomol..

[91] Vessey S., Vessey K.B. (2007). Linking behavior, life history and food supply with the population dynamics of white-footed mice (*Peromyscus leucopus*). Integr. Zool..

[92] Wolff J.O., Schauder E.M., Daniel Edge W. (1997). Effects of habitat loss and fragmentation on the behavior and demography of gray-tailed voles. Conserv. Biol..

[93] Ostfeld R.S., Keesing F. (2015). Is biodiversity bad for your health?. Ecosphere.

[94] Allan B.F., Keesing F., Ostfeld R.S. (2003). Effect of Forest Fragmentation on Lyme Disease Risk. Conserv. Biol..

[95] Stafford K.C. (2004). Tick Management Handbook. The Connecticut Agricultural Experiment Station, 71. http://www.ct.gov/caes/lib/caes/documents/special_features/tickhandbook.pdf.

